# A Multicenter Qualitative Stakeholder Evaluation of the Hospital-Based Violence Intervention Programs in the Los Angeles County Safety-Net Healthcare System

**DOI:** 10.1007/s11524-026-01059-1

**Published:** 2026-03-26

**Authors:** Zachary E. Thompson, Barbara Turner, Jennifer Laughter, Adrian Yen, Sid S. Ganesh, Shamsher Samra, Vincent Chong, Sumala Haque, Juan Garibay, Walter Afable, Sarah Axeen, Clemens Hong, Paul Giboney, Lee Plantmason, Mirna Romero, Jennifer Zuniga, Teresa Pinedo, Laura Solano, Joshua Koa, Damon Clark, Tony Kuo, Denise Villamil, Arcelia Tavarez, Todd Schneberk

**Affiliations:** 1 Keck School of Medicine, University of Southern California, Los Angeles, CA USA; 2 Gehr Center for Health Systems Science and Innovation, USC Keck School of Medicine, Los Angeles, CA USA; 3 Office of Violence Prevention, Department of Public Health, Alhambra, CA USA; 4 David Geffen School of Medicine, University of California Los Angeles, Los Angeles, CA USA; 5 Harbor-University of California Los Angeles Medical Center, Carson, CA USA; 6 Rancho Los Amigos National Rehabilitation Medical Center, Rancho Research Institute, Downey, CA USA; 7 Los Angeles County Department of Health Services, Los Angeles, CA USA; 8 Los Angeles General Medical Center, Los Angeles, CA USA; 9 Soledad Enrichment Action Community Organization, Los Angeles, CA USA; 10 Department of Family Medicine, David Geffen School of Medicine at UCLA, Los Angeles, CA USA; 11 Department of Epidemiology, UCLA Fielding School of Public Health, Los Angeles, CA USA; 12 Population Health Program, UCLA Clinical and Translational Science Institute, Los Angeles, CA USA; 13 Southern California Crossroads Community Organization, Lynwood, CA USA

**Keywords:** Social determinants of health, Violence prevention, Implementation science, Hospital-based violence intervention

## Abstract

Violent injury is a leading cause of death and non-fatal injury among individuals aged 5 to 35 in the USA, with reinjury rates ranging from 29 to 37%. This study explores the barriers and facilitators to an intervention designed to prevent violent reinjury, the Los Angeles County Hospital Violence Intervention Program (LAHVIP), through key informant interviews. This qualitative study evaluated LAHVIP through interviews with 16 stakeholders from three hospitals and their community-based organization (CBO) partners. Interviews were coded into themes based on Braun and Clarke’s methodology. Data were analyzed within the RE-AIM QuEST framework. Our results suggest that stakeholders were confident in the ability of LAHVIP to recruit patients engaged at the hospital. They also felt patients benefited from the mentorship provided by the program. Several barriers impeded program effectiveness, however, including poor integration between LAHVIP and their partner hospitals, as well as funding constraints and consequent staffing shortages. The limitation of resources in Los Angeles was also a constraint as patients had to wait several months before the services HVIP had connected them with were provided. Key factors for successful implementation included strong patient engagement, effective referrals, and the vital role of community health workers (CHWs) with lived experience. Barriers such as poor coordination between hospitals and community-based organizations, limited CHW staffing, lack of stable housing resources, and staff turnover hindered program effectiveness. Future efforts should focus on shared funding responsibility, enhanced hospital staff training, and better integration of workflows and technology to ensure long-term success.

**Supplementary Information:**

The online version contains supplementary material available at 10.1007/s11524-026-01059-1.

## Introduction

Violent injury is among the top five causes of death for individuals aged 5–35 years old, and among victims of violence, risk of reinjury ranges from 29 to 37% [1–6]. Reinjury results in repeated need for urgent medical care and can result in death [7, 8]. Hospital-based violence intervention programs (HVIPs) attempt to reduce community violence by reducing reinjury rates of individuals in cycles of violence, including domestic violence. Specifically, HVIPs link patients with services that address social determinants of health during their hospitalization [7, 9].

There are two models of HVIPs, hospital-based and hospital-linked, that are used to provide these services. Hospital-based HVIPs are operated by hospitals directly and provide direct services in addition to referrals for outside resources. Hospital-linked programs are operated by community-based organizations (CBOs) contracted by hospitals or hospital systems. Both models employ community health workers (CHWs), who often have experience with violence, to meet with patients during or shortly after their hospitalization.

Once enrolled in the program, patients are offered case management, longitudinal peer mentorship, and support acquiring resources. Supportive resources include housing, legal, career training, social-emotional learning classes, mental health treatment, food resources, securing social security disability benefits, and applying for victims of crime assistance funds among others. Additionally, CHWs accompany patients to appointments or contact providers on their behalf.

A systematic review of HVIPs reported a variety of benefits for participants, but methodological limitations compromise this evidence [10–12]. To our knowledge, a multicenter qualitative examination of HVIPs’ ability to engage victims of violence and provide them with needed services has not been conducted.

This study aimed to examine implementation of LAHVIP, one of the largest consortia of HVIPs in the nation, which is organized by the Los Angeles County Department of Health Services. We conducted key informant interviews with diverse front-line providers and leadership across three LAHVIP sites including one hospital-based and two hospital-linked programs. Recruitment sites included two large trauma hospitals, one regional rehabilitation center, and multiple community-based organizations (CBOs). This qualitative study employed the RE-AIM QuEST (Reach Effectiveness Adoption Implementation Maintenance Qualitative Evaluation for Systematic Translation) framework to examine barriers and facilitators to LAHVIP functionality.

## Methods

### Study Setting and Program Overview

This qualitative research project evaluated barriers and facilitators to the implementation of LAHVIP based on interviews of administration and staff at three hospitals and their CBO partners. Three Los Angeles County Department of Health Services (DHS) hospitals were included: Harbor-UCLA Medical Center, Los Angeles General Medical Center, and Rancho Los Amigos National Rehabilitation Center. The research arm of Rancho Los Amigos National Rehabilitation Center, The Rancho Research Institute, was also included. The two CBOs included were Southern California Crossroads (Harbor and Rancho) and Soledad Enrichment Action (LAGMC). Both CBOs in this study were externally funded in addition to being subcontracted to DHS organizations.

Recipients of LAHVIP services were aged 18 or older and spoke Spanish or English. Victims of interpersonal violence were identified by providers at presentation to the emergency department or during hospitalization for interpersonal violence that encompassed physical assault, sexual assault, stabbing, gunshot wounds, or other interpersonal harm. Rates of referral and engagement were similar across the three sites (data available from the authors). Enrolled patients are contacted by CBOs weekly for the 6-month program.

### Interviewers and Study Participants

To investigate barriers and facilitators to the LAHVIP, project team members, including two research assistants (LS, JL) and one medical student (ZT), conducted key informant interviews with CBO executives, administrators, and CHWs as well as hospital executives, administrators, and staff. Interviewers either completed online training or had prior experience conducting qualitative interviews. Multiple participants had professional relationships with study team members. All participants were informed of the purpose of this research study (Appendix 2).

**Fig. 1:**
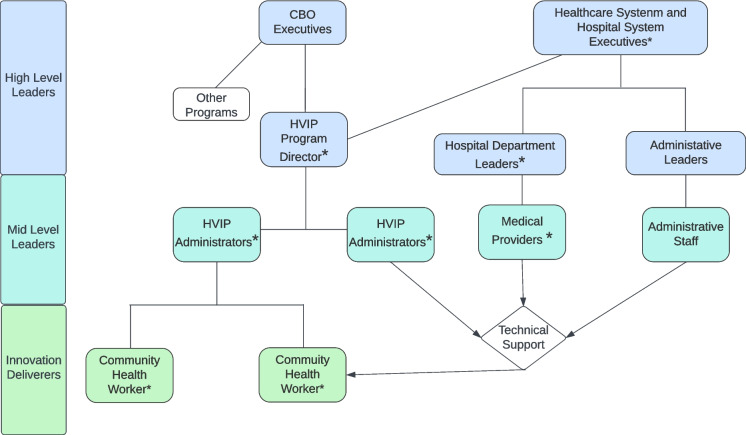
Simplified organizational chart of the Los Angeles Hospital–based violence intervention programs. Asterisks mark program roles held by individuals interviewed in this study. Arrows indicate the provision or receipt of technical support

By design, these individuals were selected to represent diverse roles within the LAHVIP organizational structure (Fig. 1). All 19 individuals known to be involved with the LAHVIP across all collaborating organizations were contacted by email and asked to agree to a 30-min interview. Informed consent was obtained by telephone prior to the start of the interviews. Of 19 stakeholders contacted, 16 completed interviews with team members (JL, LS, and ZT) from February 9, 2023, to November 30, 2024. Recruitment was concluded once team members reached consensus that data saturation had been reached.

### Interview Guide

Interview questions were drafted by study team members (TS, BT and LS) to examine facilitators and barriers to diverse program processes and functions. Draft questions were reviewed by staff of participating CBOs and hospitals for relevance, clarity, and completeness. The final interview guide (Appendix 1) asked about the following: patient engagement; opportunities to improve the hospital-CBO collaboration; challenges to implementation from the perspective of the interviewees’ organization; access to needed patient services; general attitudes about LAHVIP; and opportunities to reduce violence in the community.

### Qualitative Analysis

Interviews were conducted through Zoom and recorded with permission. Interviews were then transcribed. Transcriptions were stored on a secure password-protected server and assigned a study number. Personal identifiers were removed. Transcriptions were examined using ATLAS.ti and independently coded by AY, JL, and ZT based on the thematic qualitative methodology suggested by Braun and Clarke [13–15]. Coding was completed separately by at least two coders. Disagreements were resolved by discussion or by a third coder if required. Member checking, in which transcripts and themes were assessed by a selected participant, was used to validate our results.

Emergent themes were identified by the research team. Thematic analysis was employed within constructionist and contextualist paradigms. An iterative process was conducted to rework themes until there was unanimous agreement that themes captured the majority of the data. Themes were classified according to five general domains of the RE-AIM QuEST Framework: (1) Is the intervention reaching the target population? (2) To what extent are those targeted to deliver the intervention participating? (3) To what extent was the intervention consistently implemented? (4) Does the intervention accomplish its goals? and (5) To what extent did the intervention become part of routine organizational practices and maintain effectiveness?

### Ethics Statement

Verbal informed consent was obtained and documented prior to interviews. Verbal consent was required due to interviews being conducted remotely. Study design, materials, protocols, and all evaluation plans were approved by the University of Southern California Social Behavioral IRB.

## Results

Interviews were completed with 16 participants: four CHWs, four CBO administrators, two healthcare executives, two CBO executives and four medical providers identified as program champions (as shown in Table 1). Three additional participants were unable to participate due to scheduling conflicts. Key themes of the qualitative analysis consistent with the RE-AIM QuEST framework are summarized with quotations from interviewees.

**Table 1:** Respondent characteristics

Participant demographics	*N* = 16
Age 30–39 40–49 50–59 60–69	7 5 3 1
Sex Male Female	10 6
Role HVIP CHWs CBO administrative staff Medical providers Healthcare executives CBO executives	4 4 4 2 2

### Reach

#### “CHWs as Credible Individuals”

The firstRE-AIM QuEST domain evaluated was the ability of LAHVIP to reach intended recipients of the program. Because CHWs had personal experience with community violence, they felt effective overcoming the mistrust of health care institutions that many participants had. This is consistent with prior literature [16, 17].

I think that’s one of the biggest pluses in our programs. When we target our patients, they see us in the same situation that they’re in at the moment. And a lot of times that carries a little bit more weight. - CHW

There’s always hesitancy to work within any kind of system because there’s such distrust. So, I think we’ve set up a program where people from the community are the ones that can reach out [to patients]. - CBO Executives

#### “Funding Instability”

Regarding barriers to participation in the LAHVIP, interviewees highlighted that funding constraints and consequent CHW staffing shortages hindered CBOs’ ability to meet the needs of victims of violence in this large metropolitan area. Recent reviews have noted the importance of supporting CHWs in their critical role in HVIPs, yet our results show they are under strain [17, 18].

There’s over 700 patients who come into the hospital and we only have four or five case managers, and the capacity is like 20, 30 clients per caseload. - CBO Administrator

If we were able to staff a 24-hour team, then I think we would have a higher enrollment rate when it comes to patients that maybe come in certain hours. - CBO Executive

#### “Difficulty Contacting Participants After Discharge”

Interviewees also emphasized the challenge of reaching patients following hospital discharge, especially those with unstable housing or inconsistent access to a cell phone. These barriers to recruitment have been noted within HVIPs and other hospital-based programs [16, 17, 19, 20].

So it’s kind of hard to follow up unless they have an emergency contact, but a lot of the times I’ve noticed that when I do follow up with one of their emergency contacts, they’re like, “Oh, well, he’s lost or she’s lost. You’re not going to be able to help.‬” - CHW

Usually… a lot of clients that their phones get disconnected, and we have to do a lot of chasing after that. - CBO Administrator

### Delivering the Intervention

#### “Persistent Commitment Is Required to Ensure the Receipt of Support Services”

The intensity of HVIP programming was noted to be directly related to its perceived success. Previous investigations have shown HVIPs are able to meet over 50% of enrolled clients’ needs [17].

We ensure that you get the services that you requested, and when you can’t make a phone call, we’re following up on your behalf.” - CBO Administrator

I think everyone is aligned on our passion. Everyone is here and does work because we think our patients need more and deserve better. - Medical Provider

It’s a process. It’s never one or two and three episodes and you’re done. It’s a process that may take a while to get people out of the situations they’re in. - Healthcare Executive

#### “Hospital Partners Are Unaware of the HVIP Program”

Interviewees noted a lack of engagement with HVIP by clinical teams or staff at collaborating hospitals, who were often unaware of the program’s existence.

You might be working with one group of social workers that's totally behind what hospital violence intervention is. But the next day you might encounter somebody… that has no understanding really of what these programs are. - CBO Administrator

I think we’ve been struggling with getting information from whether it was…the clinical social workers or the staff that's in the area to provide information.- CHW

Our peer counselors are finding [eligible patients] first versus being referred. And so, it’s a lot of manual work. So that's holding us back. - Healthcare Executive

### Consistency

#### “Importance of Initiating the Program Immediately” and “Need for Continuous Active Outreach”

The third domain addressed the consistency of delivering the LAHVIP intervention to victims of violence. The intensity of the program was again emphasized.

We work well providing service to patients when we’re able to enroll them into the program and start working on their healing process immediately.‬ - CHW‬‬‬‬‬‬‬‬‬‬‬‬‬‬

We don’t wait for the clients or even patients to come to us. We go to them wherever they’re at. And that has gained a lot of trust with [our organization]. - CBO Executive‬‬‬‬‬‬‬‬‬‬‬

#### “Funding Instability” and “Frequency of Staff Turnover”

Funding for HVIP programs is seldom discussed in the literature; however in our interviews, there were frequent discussions of funding issues and its consequences including staff turnover.

I think the challenge with a lot of these programs is that there's so much turnover with staff.- CBO Administrator

If you support programs adequately where the programs don’t have to worry about funding every year or every two years in some cases, programs are able to focus on the actual work. - CBO Administrator

### Effectiveness

#### “HVIP Is Effective in Engaging Victims of Violence”

The fourth domain, effectiveness, was associated with the theme: “HVIP is Effective in Engaging Victims of Violence.”‬ Similar to prior literature, the role of CHWs as role models was an important aspect of HVIPs’ effectiveness [17, 21].‬‬‬‬‬‬‬‬‬‬‬‬‬‬‬‬‬‬‬‬‬‬‬‬‬‬‬‬‬‬‬‬

At times, folks are young and healthy, and they don’t have other things going on. We kind of frame it as service provision, but a lot of times, it’s not the services that people need. It’s kind of the accompaniment.” - Medical Provider

I think success is probably defined by the ability of a program and its staff to actually engage with victims of interpersonal violence.‬ - CBO Administrator

#### “Hospital Partners Are Unaware of the HVIP Program”

Implementation, specifically the ability of HVIPs to facilitate the provision of services, was compromised during periods when the hospitals and CBOs were not well integrated:

There’s a lot of time it takes to onboard new case managers to be contract [hospital] employees. And honestly, this is a huge headache.‬ - Medical Provider‬ ‬‬‬‬‬‬‬‬‬‬‬‬‬‬‬‬‬‬‬‬‬‬‬‬‬‬‬‬‬‬‬‬‬‬‬‬

However, there isn’t one identified person for the agency at [hospital] in the HR department that works directly with nonprofits. So being able to streamline that is sometimes a little bit difficult.‬ - CBO Administrator‬‬‬‬‬‬‬‬‬‬‬‬‬‬‬‬‬‬‬‬‬‬‬‬‬‬‬‬‬‬‬‬‬‬‬

Getting [Case managers] into the inpatient setting... it is a challenge because there might be a lot of pushbacks because you're unknown, a lot of people don’t know what’s going on‬.- Medical Provider

Implementation was further hampered by the victims’ social determinants of health including a constellation of challenges such as poverty and homelessness, which is consistent with prior investigations [20].

Some of the patients need clothes, need shoes, and sometimes when there’s not enough funding, to be honest, we’re scraping between everybody and pitching in to get that kid some shoes, because he has a hole in his shoe.‬ - CHW

‬‬‬‬‬‬

[Unhoused patients], sometimes, unfortunately, they get assaulted in the shelter setting… I’ve seen one of the patients deny resources from a social worker. They’re like, ‘I don’t need it,’ because they’ve never seen it go places.‬ Or they’ve been places and they’ve been let down, a lot of the times. - Medical Provider‬‬‬‬‬‬‬‬‬‬‬‬‬‬‬‬‬‬‬‬‬‬‬‬‬‬‬‬‬‬‬‬‬‬‬‬‬‬‬‬‬‬‬‬‬‬‬‬‬‬‬‬‬‬‬‬‬‬‬‬

### Maintenance

The last dimension of the framework related to sustainability or maintenance of LAHVIP. The stakeholders primarily described challenges to achieving this goal with one notable success.

Because we didn’t get enough money for this fiscal year compared to last fiscal year, so we had to let some folks go, even though the folks were amazing.‬ - Medical Provider

And the other problem is that community-based service providers, there’s a high turnover there…In part because of the same issues that we are dealing with in terms of sustainability.‬ - CBO Administrator‬‬‬‬‬‬‬‬‬‬‬‬‬‬‬‬‬‬‬‬‬‬‬‬‬‬‬

Alternatively, the one HVIP program in this analysis that received funding more directly from its hospital system, rather than through external grants, noted benefits:

They actually provided some of the first funding through that office to establish some of the pilot [HVIP] and we’ve grown because of that support and advocacy.- CBO Administrator

## Discussion

This qualitative study revealed key features of effective implementation of HVIPs as well as challenges to the collaborative care model used in some evaluated programs. The RE-AIM QuEST framework provided an evidence-based structure for our analysis [19, 22–24].

The first domain of the framework addressed patient engagement. Our results indicated that CHWs felt well prepared to engage patients because of their lived experience within their community. While our data is limited by our sample size of CHWs, these results are supported by prior research noting the importance of lived experience [17]. Knowing their communities and the way violence exists within them facilitated efforts to engage and support victims of violence with empathy. Additionally, while lived experience is important, it by itself is insufficient to make effective CHWs, as training, cultural competence, and communication skills, among other attributes, are equally important [25].

While CHWs were well equipped to engage victims of violence, LAHVIP was only able to enroll a fraction of the thousands of victims of violence admitted to the three participating major hospitals serving a vast urban area. The lack of stable housing and contact information within this population presented a significant challenge to enrollment. These issues are not unique to these programs. Another urban program aiming to enroll individuals with non-fatal assaultive firearm injuries succeeded in engaging only 12% of eligible individuals [20]. Proposed initiatives to support unhoused persons may serve to reduce violence and facilitate future efforts to address socio-emotional challenges of victims of violence [20, 26, 27].

Another barrier to enrollment was lack of familiarity with the program among hospital staff. The HVIP model relies on rapid referral by hospital staff. This method of identification was compromised by poor awareness and engagement among hospital staff particularly within the hospital-linked programs. While efforts were made to educate staff about HVIP, this was not enough to improve incentives for staff to engage. More top-down support from the hospitals is likely needed to encourage hospital staff to participate.

A third domain of the framework was consistency of delivering the intervention. CHWs and other stakeholders emphasized their commitment to the HVIP model and its mission, which contributed to consistency. To accomplish the goal of assisting patients, CHWs emphasized their personal efforts. For example, they scheduled appointments with service providers in addition to physically accompanying patients to appointments to ensure that the providers’ plans were carried out. CHWs acknowledged that at times they helped patients using their private money or resources. This level of commitment is helpful but demonstrates gaps in other aspects of program implementation such as funding. Furthermore, the use of personal resources could lead to staff burnout.

Consistent delivery of LAHVIP was also facilitated by program champions such as physicians, nurses, or other staff at participating hospitals. Champions identified eligible patients after admission for referral to CBOs and advertised the program to colleagues. A recent review reinforced the key role of clinical champions in the care and support of victims of domestic violence [28]. Yet the champion role is often informal and uncompensated. As a result, their commitment is much less certain and they are more predisposed to burnout.

Process challenges, for example, lack of CHW access to the hospital’s electronic medical record, were another barrier to consistency for the hospital-linked programs in particular. The HVIP model entails engaging patients admitted day or night, as patients often have limited hospital stays. Each hospital would ideally have one CHW onsite at all times, but this was not always achieved. For example, at one site there was a period when only one CHW had access to patients, as other CHWs were involved in a prolonged and unclear credentialing process.

This challenge was made worse by frequent staff turnover. Turnover of CBO staff and CHWs can lead to the loss of personnel with the relationships, skills, and knowledge to effectively navigate the complexities of the local resource environment. New personnel requires substantial training using a standardized curriculum as well as interpersonal skills training. Therefore, programs often lose well-trained staff during times of funding cuts, only to spend considerable resources training new staff when funding returns. Because of the tenuous nature of funding, one group has suggested performing a risk stratification for the likelihood of future violence to identify those in most need of HVIP services [29]. Such an initiative could ensure a more intensive and consistent delivery of services but may miss individuals who could benefit greatly from the program.

Finally, RE-AIM QuEST addressed if the intervention ultimately achieved its goals: reduction in violent reinjury and the provision of services or resources. Both hospital and CBO interviewees noted that enrolled patients received needed services and support. Moreover, the CHWs served as positive role models who reinforced the value of programs. For example, CHWs helped individuals stay away from illicit or gang-related activity.

In this qualitative study, patients in the program were not interviewed, and therefore we did not obtain information on whether enrolled individuals were able to find housing or other social support. We were also unable to assess how effective patients felt the programs were in preventing future violence. However, the ability of HVIPs to reduce the severity and frequency of violent activity is evidenced by clinical trials which demonstrated that HVIP participants have significantly less prison time than controls [12]. A systematic review of HVIP-like programs for victims of violence in the emergency department also reported statistically significant improvements in multiple measures of subsequent violence [30]. Still, the ability of programs may be limited in terms of their effectiveness to address housing and mental health [16]. It is essential to perform future studies that more comprehensively evaluate quantitative outcomes such as these measures of violence for the LAHVIP.

The success of the HVIP model relies on adequate funding for this program. Everytown for Gun Safety estimated that in a mid-sized city, funding for an HVIP would require $10,800 per participant; in a large city with thousands of victims of violence this would cost millions of dollars [31]. However, the funding is not only for the HVIP itself. Stakeholders often discussed substantial waitlists and delays between applying for services or resources and patients receiving them. Many services were at county or municipal levels and not under control of the HVIP program staff, which served as a source of frustration. These delays led patients to drop out of the program.

Hospital-based violence intervention programs require the close collaboration of hospitals and community service providers to function. As prior qualitative investigations have noted, the quality of this collaboration is key to facilitating the linkage of needed resources, services, and support to patients [21].

### Limitations

This qualitative study has several limitations. First, LAHVIP was implemented in one of the nation’s largest cities and our findings may have limited external validity in rural areas due to the program’s urban setting. Nevertheless, to our knowledge, this is the first qualitative study of implementing an HVIP across multiple hospitals and CBO organizations. Second, this analysis did not examine the experiences of patients served by this program. Third, our findings may be influenced by interviewer positionality, participant awareness of our research aims, and relationships between study team members and participants. Lastly, this study is limited by its qualitative methodology and its usefulness is primarily limited to hypothesis generation. We hope, however, it will help guide other systems in developing or maintaining HVIPs.

### Implementation Opportunities

Implementation barriers in LAHVIP persist largely due to a lack of integration between hospitals and community-based organizations (CBOs). To improve coordination, several changes could be made. First, shared funding responsibility between hospitals and CBOs would help alleviate financial pressures on programs and incentivize better collaboration. This would also address the current instability in CBO staffing, which is heavily reliant on short-term grants. Second, coordinated information campaigns for hospital staff would improve cooperation between case managers and providers. Standardizing workflows and integrating technology—such as shared patient data systems—would streamline patient enrollment and ensure more consistent care. To further enhance integration, HVIP workers should be co-located within the hospital teams, making them a core part of the healthcare infrastructure, similar to other community health workers. This co-location model is a best practice which has already been implemented in some programs [21]. Lastly, patient enrollment could be enhanced by using novel communication strategies such as social media to stay in contact with individuals without access to phones. These changes would not only improve program efficiency but also foster hospital buy-in, ensuring that HVIPs are seen as vital members of the healthcare system, ultimately improving patient outcomes.

## Conclusion

This qualitative study of LAHVIP revealed key strengths and challenges in implementing a collaborative care model between hospitals and community-based organizations to support victims of violence. The findings underscore the importance of patient engagement, the critical role of community health workers with lived experience, and the need for hospital staff buy-in to ensure effective referral and longitudinal care. Challenges such as insufficient staffing, lack of stable housing resources, and inconsistent program delivery due to staff turnover hinder the program’s effectiveness. Despite these obstacles, stakeholders expressed a strong commitment to the program’s mission, and early evidence suggests that victims enrolled in the program received valuable services. Future directions should focus on securing adequate and sustainable funding, addressing logistical barriers like access to medical records and expanding the program’s reach through better infrastructure for longitudinal care. Further quantitative outcome evaluations are needed to assess the program’s long-term impact, particularly in reducing violence recurrence and addressing socio-emotional needs.

## Supplementary Information

Below is the link to the electronic supplementary material.

ESM 1 (DOCX 16.6 KB)

## Data Availability

Due to the sensitive nature of the qualitative data and the conditions of Institutional Review Board approval, full interview transcripts are not publicly available. De-identified data supporting the findings of this study may be shared upon reasonable request to the corresponding author, subject to IRB approval and data use agreements.
