# Supplementary material for: A Multicenter Qualitative Stakeholder Evaluation of the Hospital-Based Violence Intervention Programs in the Los Angeles County Safety-Net Healthcare System
Source: J Urban Health. 2026 Mar 26;103(2):443–53. doi: 10.1007/s11524-026-01059-1 (PMC13235664; doi:10.1007/s11524-026-01059-1)
Supplement: Supplementary file 1 — (DOCX 16.6 KB) [file 11524_2026_1059_MOESM1_ESM.docx]

Appendix A:

**Interview Introduction**

My name is ____________. Thank you for your willingness to talk to me today about your experiences with Hospital-Based Violence Intervention Programming in Los Angeles County.

Our interview will be audio recorded, and our research team will transcribe the interview. Any information collected will be de-identified and stored on a secure server. Please know that all of your responses will be kept confidential, so you can feel free to share your real opinions without fear of judgment or repercussion. Your openness and honesty will greatly contribute to the success of our study. Your name will not be shared or attached to any statement you make.

This interview will take about 30 minutes because we really value your input on how to improve this program.

**GENERAL OPENING**

Q1. Let’s start, can you tell me briefly about your current role at your organization?

How does your role relate to providing hospital based violence intervention or trauma wraparound care?

(Note to interviewer - keep brief)

Prompt: Are there challenges you have in your role in helping patients who are victims to violence?

Are there aspects to your role that you find are successful?

**Program Facilitators**

Q2. From your perspective, what are some ways that the hospital-based violence intervention program/s work well in serving patients who are victims of violence?

Probe - can you tell me more about the ways that the program benefits these patients?

**Program Barriers**

Q3. In terms of how the program works at your institution or organization, are there specific challenges that you believe may be preventing it from serving patients adequately?

Probes:

Do you feel like there is support from county programs?

Q4. Are there ways the hospital system partnership can support you better?

Q5. When considering barriers to better serving these patients, what do you believe are insurance or other barriers outside of the hospital to the program helping patients who are victims of violence?

Q6. Can you tell us about any difficulties you have in reaching patients who are victims of violence to engage in the program?

Probe – ex people who are homeless, etc

Q7. On a community level, from your perspective, what are some of the ways that the hospital-based violence intervention program/s could reduce the impact of violence in the LA community in general?

Q8. Do you have any other ideas or thoughts about how to improve violence intervention services at the program level or the community level that would like to tell us about?

If no - Those are all the questions I have today. Thank you so much for your time. Before we end the interview, I just have a few demographic questions to ask.

1. Can you please tell me the title of your current position? ______________________

2. How many years have you been in this organization?____ (months)_______ (years)

3. Which of the following describes your race?

☐ White ☐ Black or African American ☐ Hispanic/Latino/a

☐ American Indian or Alaska Native ☐ Asian or Asian American

☐ Native Hawaiian/Other Pacific ☐ Other:___________

4. What is your gender identity?

☐ Male ☐ Female ☐ Non binary/queer ☐ Prefer not to answer

5. Please select your current age range.

☐ 20-29 years old

☐ 30-39 years old

☐ 40-49 years old

☐ 50-59 years old

☐ 60-69 years old

☐ 70-89 years old

**Notes on the Interview**

Information about the setting:

Informant _____________________

Mode of Interview (Phone, Video) _____________________

Date ____________, Time _______________, Interview Length _____________________

Approach:

• Approval for taping

• Greetings

• Descriptive questions

• Exit comment
